# Patient safety in community-based mental healthcare: A systematic scoping review

**DOI:** 10.1192/j.eurpsy.2022.1615

**Published:** 2022-09-01

**Authors:** P. Averill, C. Vincent, G. Reen, N. Sevdalis, C. Henderson

**Affiliations:** 1 King’s College London, Centre For Implementation Science, Institute Of Psychiatry, Psychology And Neuroscience, London, United Kingdom; 2 University of Oxford, Department Of Experimental Psychology, Oxford, United Kingdom

**Keywords:** review, mental health care, Patient safety

## Abstract

**Introduction:**

There is limited existing research about patient safety issues in mental healthcare. A lack of evidence is particularly pronounced in relation to safety in community-based mental health services, where the majority of care is provided. To date, reviews of mental health patient safety literature have focused primarily on inpatient care settings.

**Objectives:**

This systematic scoping review will aim to identify and synthesise literature about the types of patient safety problems in adult community-based mental health settings, the causes of these problems, and evaluated safety interventions in this care context.

**Methods:**

A systematic search was conducted on 19^th^ June 2020 and refreshed on 23^rd^ October 2021, across five databases: Medline, Embase, PsycINFO, Health Management Information Consortium, and Cumulative Index to Nursing and Allied Health Literature. The search strategy focused on three key elements: ‘mental health’, ‘patient safety’ and ‘community-based mental health services’. Retrieved articles were screened at title, abstract and subject heading level, followed by full-text screen of longlisted articles.

**Results:**

In this presentation, the findings of this systematic scoping review will be described, based on synthesised literature about safety incidents, broader care delivery problems, their causes, and evaluated patient safety interventions to address these issues.

**Conclusions:**

This study will offer learning opportunities about the safety problems, contributory factors, and safety interventions in adult community-based mental health services, as described in the evidence base. Review findings will also help to ascertain gaps in existing research, which should be addressed in future studies.

**Disclosure:**

NS is the director of London Safety and Training Solutions Ltd, which offers training in patient safety, implementation solutions and human factors to healthcare organisations and the pharmaceutical industry. The other authors have no competing interests.

